# The Use of Narcotics

**Published:** 1883-05

**Authors:** 


### EDITORIAL, ETC.

THE USE OF NARCOTICS.—At the late meeting of the Maryland Medical and Chirurgical Faculty, in Hopkins Hall, Dr. G. Halstead Boyland read an interesting paper on "Hypnotism." He said that the influence of climate upon race is nowhere so forcibly manifested as in America. The various changes have left their impress upon succeeding generations, and the inhabitants of to-day are stimulated to unnatural eagerness of temperament, with intervals of excitement and apathy, resulting in the rapid rate of life of the nineteenth century and the "cramming" system of education, the outcomes of an inherent desire to have as much as possible in a short space of time. After prolonged business strain or society dissipation come restlessness and sleeplessness. The rational remedy would be regular hours, less work and more relaxation, and a natural plan of treatment, extending over weeks, months or years. But the craving for the unnatural will not brook such delay, and all classes resort to the quicker methods of laudanum, hydrate of chloral, bromide of potash, morphia or opium. Those using them have, in many cases, become acquainted with them through physicians and druggists.

There being no State or municipal law restricting the sale of hypnotics by chemists and druggists, the public are at liberty to purchase any quantity they please, or the requisite drachm of laudanum may be had at the grocery store. This habit of narcotics is thus sown broadcast, and is largely on the increase. Bright's disease, paralysis, agitans, chronic nervous exhaustion, cerebral anæmia and irritability have been directly traced to their use. Patients addicted to them often alternate them with alcoholic drinks, and women take them to restore quiet and tone to the shattered nervous system after late hours, dancing and worry. The object is never attained, for the greater the quantity of the drug used, and the oftener repeated, the worse the condition of the patient. Finally, nothing is left to go upon, and the general system, utterly wrecked, is invaded by organic diseases of the brain, lungs, heart, liver or kidneys.

Perhaps the majority of these troubles can be traced to the use of patent medicines and nostrums given during infancy and childhood. The quantity taken at first is quite insignificant, but soon loses its effect, and has to be increased, until it becomes enormous.

A number of interesting cases were given. Dr. Boyland says: "The statement that chloral is to be found in the work-boxes and baskets of nearly every lady in the 'West End' may be well supplemented by another on this side of the Atlantic, to the effect that the ladies resort to their chloral draughts at night, or their bromide during the day, a supply of these things being kept constantly on hand. The soda-water fountains find many who take 10 to 30 grains of bromide of potash at greater or less intervals. Bromide of potash, if persisted in, will reduce the system to such a state as to be easily invaded by anæmic cerebral spinal disorder." The paper shows that hypnotism is never permanently cured, and says "if the law prohibited the sale of hypnotics to the laity, the number of suicides and suicidal attempts by laudanum and morphia would be reduced to a minimum, but as long as the indiscriminate traffic in them is permitted, uncontrolled by physicians and in the absence of State or municipal law, just so long will there be annually an increase in the promiscuous use of bromide of potash, chloroform, dydrate of chloral, laudanum and morphia, and a proportionate amount of vice, unhappiness, crime and disease.

---

### TREATMENT OF FRACTURED JAW.

TO THE EDITOR AMERICAN JOURNAL OF DENTAL SCIENCE.

